# Modelling Interfaces in Thin-Film Photovoltaic Devices

**DOI:** 10.3389/fchem.2022.920676

**Published:** 2022-06-21

**Authors:** Michael D. K. Jones, James A. Dawson, Stephen Campbell, Vincent Barrioz, Lucy D. Whalley, Yongtao Qu

**Affiliations:** ^1^ Department of Mathematics, Physics and Electrical Engineering, Northumbria University, Newcastle Upon Tyne, United Kingdom; ^2^ Chemistry – School of Natural and Environmental Sciences, Newcastle University, Newcastle Upon Tyne, United Kingdom

**Keywords:** kesterite Cu_2_ZnSnS_4_ thin films, CZTSSe, CZTS, interface, modelling, photovoltaic, thin-film, device

## Abstract

Developing effective device architectures for energy technologies—such as solar cells, rechargeable batteries or fuel cells—does not only depend on the performance of a single material, but on the performance of multiple materials working together. A key part of this is understanding the behaviour at the interfaces between these materials. In the context of a solar cell, efficient charge transport across the interface is a pre-requisite for devices with high conversion efficiencies. There are several methods that can be used to simulate interfaces, each with an in-built set of approximations, limitations and length-scales. These methods range from those that consider only composition (e.g. data-driven approaches) to continuum device models (e.g. drift-diffusion models using the Poisson equation) and *ab-initio* atomistic models (developed using e.g. density functional theory). Here we present an introduction to interface models at various levels of theory, highlighting the capabilities and limitations of each. In addition, we discuss several of the various physical and chemical processes at a heterojunction interface, highlighting the complex nature of the problem and the challenges it presents for theory and simulation.

## 1 Introduction

Energy converters, such as solar cells, re-usable batteries and fuel cells, are a key ingredient for achieving the target of net-zero carbon by 2050. With less than 30 years until this deadline, there is a strong emphasis on accelerating the development of new materials and technologies with better performance (for example, batteries with higher energy densities) or allowing access to new markets (for example, product-integrated photovoltaics). Computational modelling can be used to predict device performance without synthesis, fabrication or characterisation, guiding experimental efforts to pursue only the most promising new designs and ultimately reducing the time from materials discovery to technology deployment. (Tabor et al., 2018).

**FIGURE 1:**
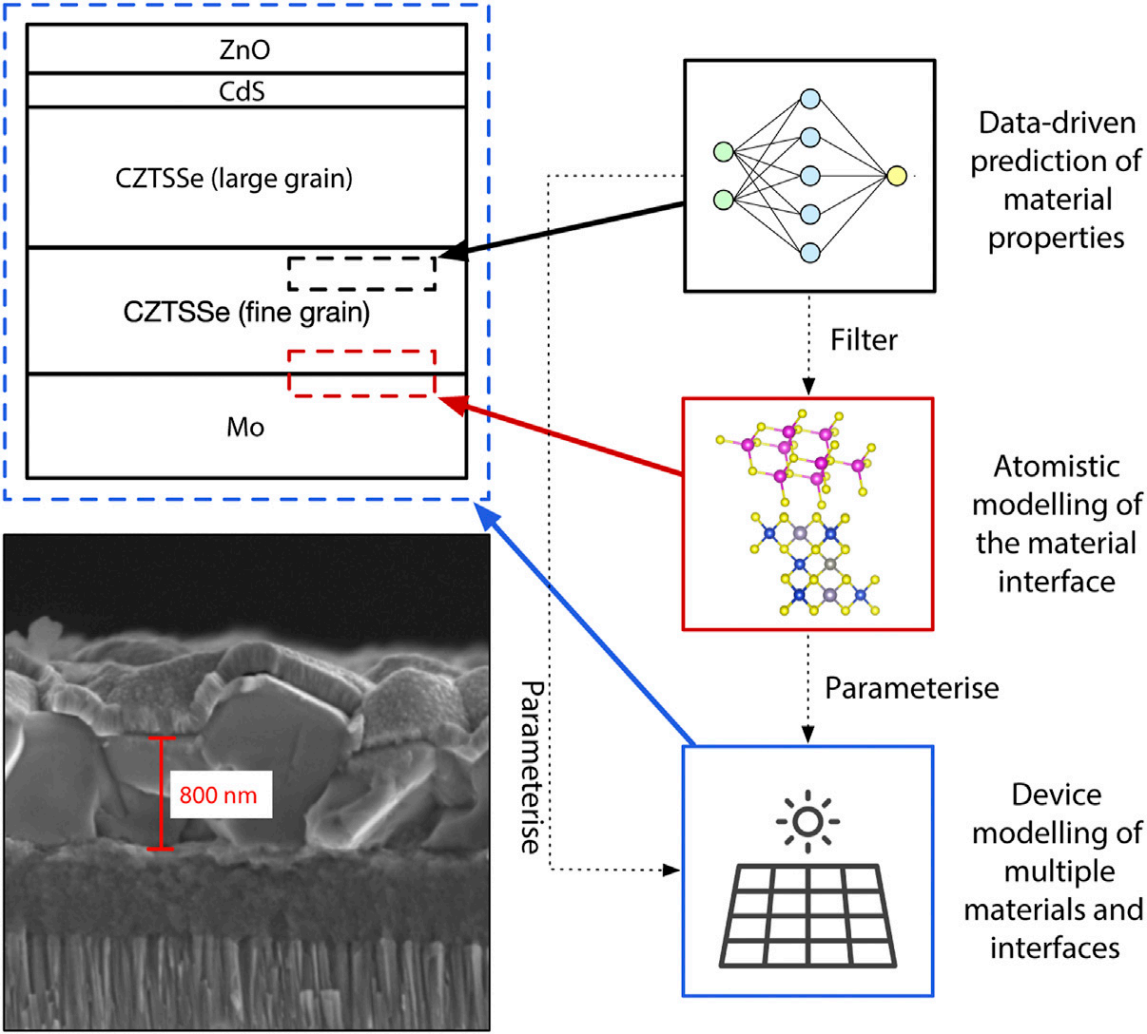
Image and schematic outlining the multi-layered structure of a typical Cu_2_ZnSn(S,Se)_4_ (CZTSSe) solar cell and the various approaches to interface modelling. The bottom left image is a Scanning Electron Microscope (SEM) image of a CZTSSe thin film photovoltaic device fabricated from nanoparticle inks. The length scale is indicated in red. The top left is a schematic of the same device, shown for clarity. The three boxes on the right outline three complimentary approaches to interface modelling: 1) (black boxes) data-driven approaches for predicting the properties of a single interface material; 2) (red boxes) atomistic modelling of the interface between two materials; and 3) (blue boxes) continuum modelling of the complete device, including multiple materials and interfaces. The dotted arrows indicate how these models relate to one another: lower accuracy data-driven predictions can be used as a initial filtering step before higher accuracy atomistic models are applied, and both of these approaches can be used to parameterise device models. The solar cell icon is a resource downloaded with permission from Flaticon.com.

Photovoltaic (PV) cells are generally semiconductor heterostructures consisting of multiple material components. In thin-film PV devices each component has a thickness ranging from a few nanometres to tens of micrometres—a typical example of a thin-film PV device structure is shown in Figure 1. Commercialised thin-film materials include CdTe, CuIn_1−__*x*_Ga_*x*_Se_2_ (CIGS), and amorphous thin-film silicon (a-Si), whilst perovskite-silicon tandem cells are targeted for commercial production by the end of 2022. There is also research activity around an array of materials with potential for commercialisation, including Cu_2_ZnSnS_4_ (CZTS), (Liu et al., 2020), Sb_2_Se_3_, (Liu et al., 2020), SnS, (Cho et al., 2020), Cu_2_O, (Wei et al., 2012), and AgBiS_2_. (Ju et al., 2020).

The interfaces in a PV device are particularly important as they determine the carrier dynamics and so the overall device performance. (Edward et al., 1992; Franciosi and Van de Walle, 1996; Fritsche et al., 2001; Fritsche et al., 2005; Jaegermann et al., 2009; Li and Jen, 2022). To understand why this is one must first have a basic understanding of the processes underlying PV performance. Consider, for example, a solar cell with a planar structure: 1) a photon is absorbed and an exciton is created; 2) the exciton separates into an electron and hole; 3) the electron and hole travel through the absorber material; and 4) the electron and hole are extracted through an interface to their respective contact materials. The contact materials may be a metal contact, a buffer layer, a window layer, or an electron/hole transport layer. In addition, for polycrystalline materials there are additional interfaces in the form of grain boundaries. Before successfully reaching an external circuit to do useful work a charge carrier will need to traverse multiple interfaces.

There are several examples where interface engineering has improved the light-to-electricity efficiency of a device. To take one example, consider the development of CdTe solar cells. Most devices used a CdS buffer layer between the absorber material and transparent conducting oxide (TCO) until it was demonstrated in 2016 that replacing this with Mg_x_Zn_1−__*x*_O led to improved performance. (Kephart et al., 2016). The reason for this improved performance is threefold and demonstrates the variety of functions an interface material must fulfil: the electronic band alignment with the TCO can be optimised with the proportion of Mg content, Mg_x_Zn_1−__*x*_O allows for a higher temperature deposition of the CdTe which results in larger grain sizes and improved charge transport, and Mg_x_Zn_1−__*x*_O has a larger optical band gap for better ultraviolet light transmission. (Kephart et al., 2016).

The rate of charge transport across an interface is primarily dependant on the energy band alignment of the constituent materials and the defects existing at the interface, so these are key considerations when optimising device designs. However there are additional considerations including chemical compatibility, heat transfer, (Sutanto et al., 2020), the mechanical stability provided by the interface layer and the rate of intended, or unintended, ion transport. (Prabhakar and Jampana, 2011). Whilst accurate computational predictions of the properties and processes within a single bulk material are now well established, (Oba and Kumagai, 2018), capturing the complex physics and chemistry at the interface between two materials remains an on-going challenge for theory and computation. (van der Giessen et al., 2020). In particular, linking microscopic structures and processes with material function is an inherently multi-scale problem that requires a range of approaches (Figure 1).

For materials design and optimisation where the structure of a material (or materials) is unknown there are a growing number of studies using the large amount of materials data that has already been generated and is readily available, or that can be generated using high-throughput-computing. For example, machine learning models have been used to predict thermodynamically stable quaternary oxides (Davies et al., 2019) and derive accurate force fields for molybdenum metal. (Chen et al., 2017). In the context of materials discovery, this data-driven approach can be used as a lower-cost filter before applying more expensive *ab-initio* models to the candidate interface material.

For modelling microscopic processes at the interface between two materials there are a range of atomistic simulation techniques based on either quantum chemical techniques or the equations of classical mechanics. These can also be used to calculate interface and bulk parameter values for device models. The typical size of unit cell used for interface simulations will depend on the process being modelled, the level of theory used and, unavoidably, the computer time that is available. The lower limit is on the order of hundreds of atoms, which has until recently restricted atomistic interface modelling to using classical potentials for describing the atom-atom interactions. However with recent research investment into high-performance-computing and the development of more computationally efficient codes, (Nakata et al., 2020; Prentice et al., 2020; Ratcliff et al., 2020), higher accuracy *ab-initio* quantum chemical predictions for systems with hundreds or thousands of atoms are now possible.

For predicting performance at the device level, continuum-scale models—most often Poisson-drift-diffusion simulations—are used. However capturing the full complexity of the processes at an interface in a numerical or analytical model at the device-level is not feasible. Instead it is most often assumed that the rate of charge transport is determined by the band offset energy between two materials and parameters (for example, the surface recombination velocity) which incorporate other microscopic physical effects. These values can be measured by experiment, calculated using atomistic simulations, or predicted using data-driven approaches, providing a straight-forward approach to multiscale modelling.

The focus of this review is interface modelling in thin-film PV devices. In particular, we are concerned with modelling planar interfaces between inorganic or hybrid organic-inorganic materials, rather than the bulk heterojunctions typically found in organic photovoltaic (OPV) devices or at grain boundaries. We do not consider mesoscale models that are designed to bridge between the micro- and macro-scale as although there are a limited number of applications in the context of hybrid perovskite photovoltaics, (Bahrami et al., 2021), these models have been most widely used within the OPV community where device performance is highly sensitive to the mesoscopic structuring of the bulk heterojunction.

We begin the review by introducing the key concepts underlying energy band alignment, which is the most important consideration for the design and optimisation of PV devices. We then go on to identify the other key features of an ideal PV interface material. In the second half of the review we introduce several approaches for modelling interfaces in PV devices: 1) data-driven methods for interface materials discovery and low-cost predictions of material performance; 2) atomistic interface models for a higher-accuracy understanding of processes at the microscale; and 3) continuum device models for understanding the combined effects of multiple interfaces at the device level.

## 2 Energy Band Alignment in Photovoltaic Devices

Electron energy level differences at an interface play a key role in a solar cell device. Their relative alignment describes built in electric fields that occur across material junctions, indicate the preferred direction of drift currents and also suggest potential tunnelling mechanisms. This all gives a greater insight and understanding to the workings of a device and the role the interface plays.

Semiconductor-semiconductor interfaces can be characterised in two groups: homojunctions and heterojuctions. Homojunction describes an interface where the two neighbouring bulk materials are the same, though the materials are often differentiated through dopant species or densities. On the contrary heterojunctions are comprised of two different materials. The latter comprise the bulk of this review as they are the most well-studied interface type in thin-film PV devices. Later in this section a discussion relating to metal-semiconductor junctions, and their classification as Schottky or ohmic is presented.

**FIGURE 2:**
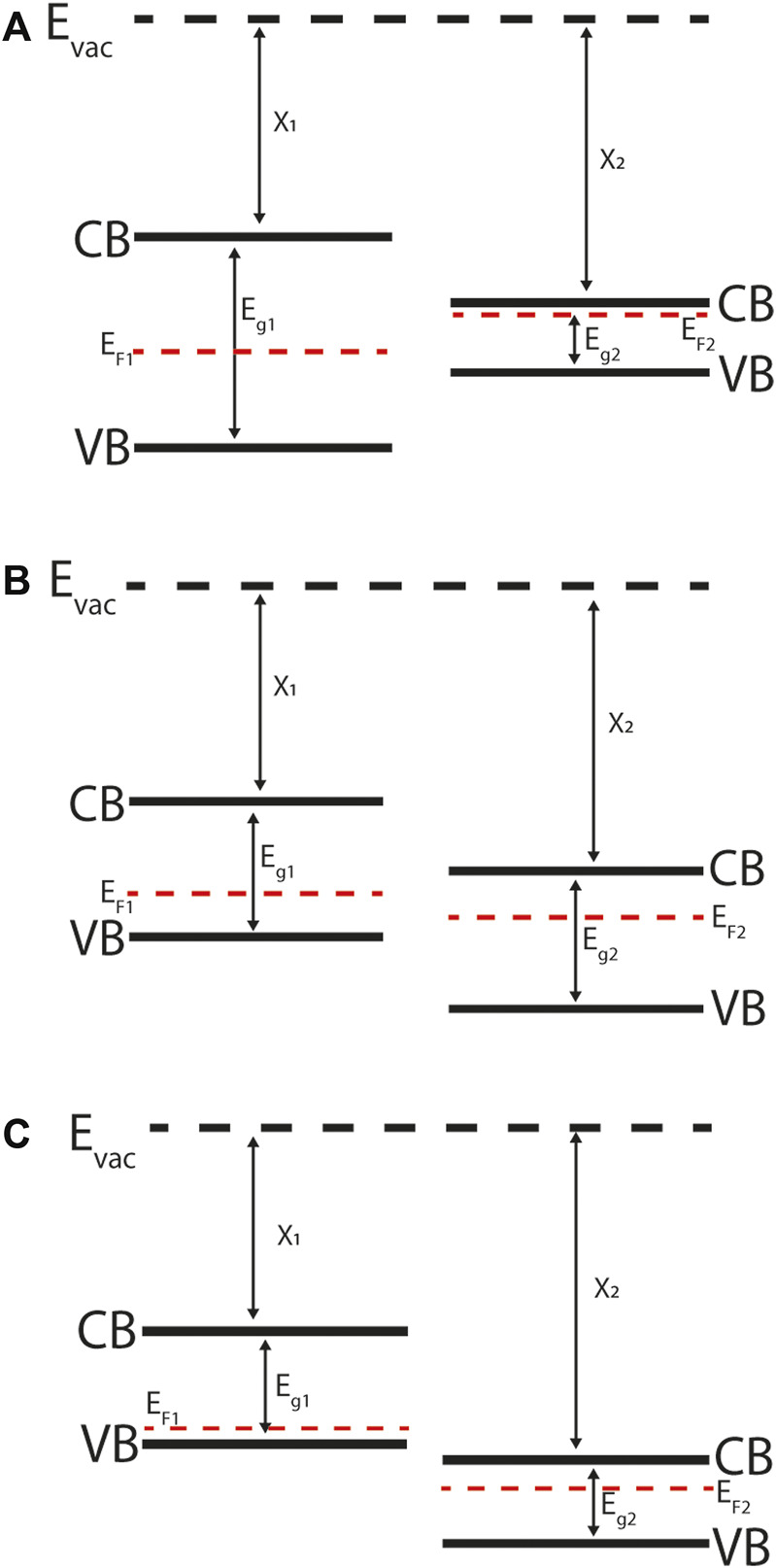
Energy band diagrams of abrupt semiconductor-semiconductor heterojunctions: **(A)** Straddling gap (Type-I), **(B)** Staggered gap (Type-II), **(C)** Broken gap (Type-III). Note that the materials are not in contact or in equilibrium with one-another, as indicated by the mismatch between their Fermi levels (*E*_F_). CB is the conduction band (minimum) and VB is the valence band (maximum) referenced to the vacuum level *E*_vac_. For quantitative predictions of band alignment the electron affinity (*χ*) and band gap (*E*_g_) are used in the Anderson rule, which assumes that the vacuum level is constant.

Our understanding of the interfaces within a device is very often summarised as an energy band diagram. Constructing energy band diagrams allows the observer to represent band-edge electron or hole energy levels, electric fields and current flow in the dimensions of energy and one-dimensional space; Figures 2, 6 are examples of energy band diagrams that will be discussed in more detail later in the paper. Many of the interesting features in energy band diagrams are at the interface region between two materials. The relative alignment at the interfacial regions of these diagrams can represent a range of features that are of importance in the design and fabrication of semiconductor devices, including: charge separation characteristics, interface bonding dynamics, interface trap states and depletion region widths.

Differences in the conduction band minimum (CBM) and valence band maximum (VBM) of neighbouring materials at an abrupt semiconductor-semiconductor heterojunction interface lead to band offsets. The type of band discontinuity depends on the electronic structure of the materials and are commonly grouped as: straddling gap (also known as Type I, Figure 2A), staggered gap (Type II, Figure 2B), and broken gap (Type III, Figure 2C). (Kroemer, 2001) Straddling gap junctions have conduction and valence band offsets of opposite sign, so that the lower CBM and higher VBM occurs in the same material. This structure promotes two-carrier processes such as radiative recombination as it is energetically favourable for the electrons and holes to occur in the same material. Staggered gap junctions have conduction and valence band offsets of the same sign, so that the lower CBM occurs in one material and higher VBM occurs in the other material, with an energy separation between the two. This structure promotes separation of electrons and holes, and so is of particular relevance to PV devices where charge separation underlies voltage generation. In broken gap junctions the CBM in one material drops below the VBM in another material and there a direct tunnelling mechanism across the interface.

For quantitative estimates of band offsets at semiconductor junctions the electron affinity rule introduced by (Anderson, 1960) is commonly used. This simple rule allows the band offset of the conduction band minima (Δ*E*_c_) and valence band minima (Δ*E*_v_) to be calculated from the bulk properties (band gap *E*_g_ and electron affinity *χ*) of the neighbouring materials. For example, consider Figure 2A where there is a straddling gap junction. In this case the band offsets are given by:

$$\Delta E_{\text{c}}=\chi_{1}-\chi_{2}$$ (1)

$$\Delta E_{\text{v}}=\left(E_{\text{g}1}-E_{\text{g}2}\right)-\Delta E_{\text{c}}.$$ (2)

Anderson’s model is based on the assumption that the vacuum level is consistent when forming junctions between two materials. This rule is often used to calculate the band offsets, followed by Poisson’s rule to calculate the shape of the band bending that occurs upon carrier density equilibration across the heterojunction interface.

**FIGURE 3:**
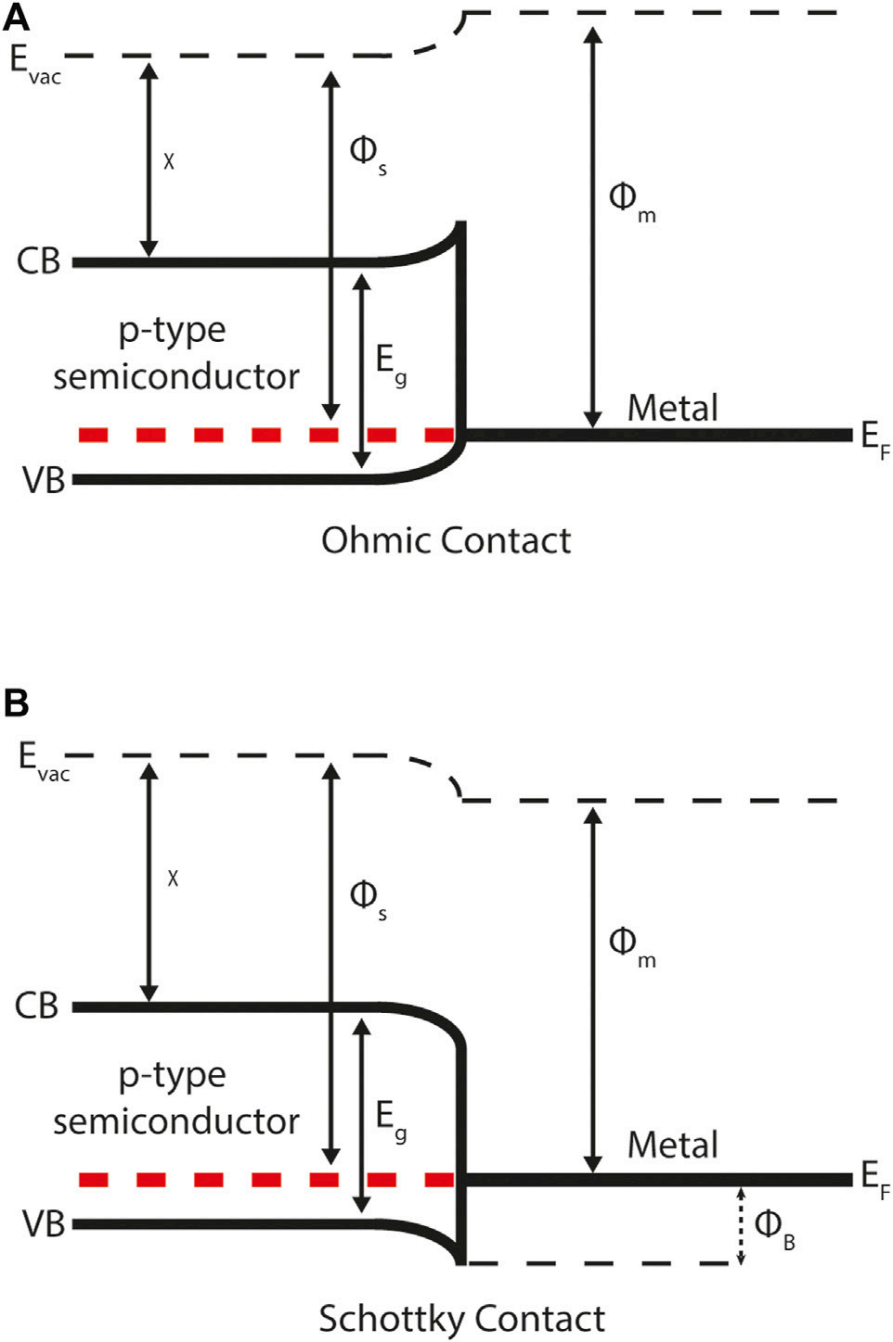
Energy band diagrams for a semiconductor-metal contact demonstrating: **(A)** ohmic behaviour **(B)** Schottky behaviour. CB and VB represent the conduction band (minimum) and valance band (maximum) respectively. Φ_B_ describes the Schottky barrier height to majority carrier flow (in this case holes) across the junction. The Schottky-Mott rule can be used to calculate Φ_B_ from the metal work function Φ_m_ and semiconductor electron affinity *χ* (both defined relative to the vacuum level *E*_vac_) and semiconductor band gap *E*_g_. In equilibrium the Fermi-level *E*_f_ is constant across the semiconductor-metal interface.

Semiconductor-metal interfaces are categorised differently given the absence of an electronic band gap in metals. They are classified as either Schottky or ohmic, depending on the difference between the semiconductor and metal work functions (Figure 3). For a p-type semiconductor with a work function greater than the metal, mobile charge will diffuse from the semiconductor into the metal and a Schottky contact will form. The resulting electrostatic field impedes majority carrier flow across the junction. For a p-type semiconductor that has a work function less than the metal an ohmic contact will form. Ohmic contacts promote majority carriers over the junction and are thus the ideal scenario for majority carrier extraction at an electrical contact.

The Schottky-Mott rule for semiconductor-metal interfaces is used to estimate Schottky barrier height Φ_B_ from the metal work function Φ_m_, semiconductor electron affinity *χ* and band gap *E*_g_. (Francis Mott, 1939) For example, consider Figure 3 where there is a p-type semiconductor in contact with a metal. In this case the Schottky barrier height is given by:

$$\Phi_{\text{B}}=\chi +E_{\text{g}}-\Phi_{\text{m}}.$$ (3)

It is important to note that both the electron affinity rule and Schottky-Mott rule can give only very rough estimates for band offsets as they ignore any chemical bonding, defect formation and electrical polarisation at the interface. These are highly idealised model that are only strictly valid in the limit of a large vacuum separation between the two materials.

For both semiconductor-semiconductor and semiconductor-metal contacts in thermodynamic equilibrium (where there is no illumination or external bias) the Fermi-level must be continuous across the interface. Physically this means that the electron or hole carriers will diffuse across the junction as a result of a concentration gradient. If the carriers were charge neutral this process would continue until there is a uniform distribution of carriers throughout the device. However, the carriers do carry charge and so they diffuse across the junction and leave behind charged ions. The charged ions form an electric field which eventually prevents further carrier diffusion. As a result of this process the conduction and valence bands exhibit band bending near the interface (Figure 3). There is no band bending in metals due to high concentrations of mobile charge carriers that can respond to the formation of an electric field.

**FIGURE 4:**
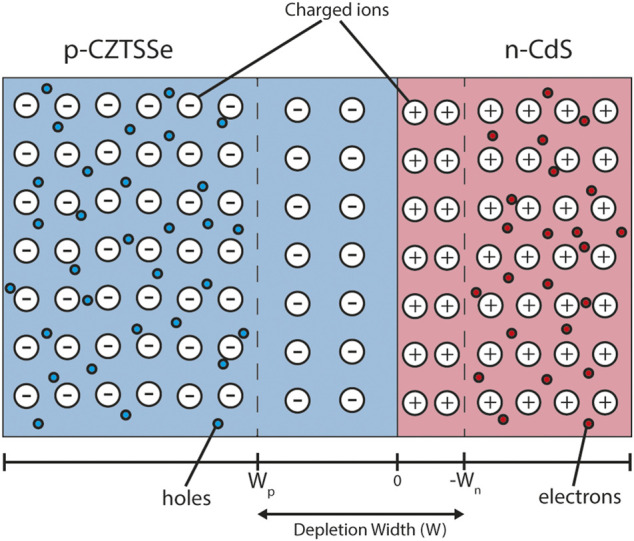
Depletion region schematic of the CZTSSe/CdS junction. The blue and red colouring represent p-type CZTSSe and n-type CdS respectively. The static charged ions—responsible for material doping—are also drawn. The depletion region does not contain any mobile charges. The black bar below denotes the material interface point (0), p-type depletion width (*W*_p_) and n-type depletion width (*W*_n_).

For a semiconductor-semiconductor p-n junction in equilibrium the majority carriers (electrons in the n-type region and holes in the p-type region) diffuse across the junction as a result of their respective concentration gradients. This region at the interface is known as the depletion region (Figure 4) as it contains a built-in electric field which removes any free charge carriers. The width of the depletion region can be calculated using Poisson’s equation and is primarily determined by the doping density and dielectric permittivity of the material.

## 3 What Makes the Ideal Photovoltaic Interface Material?

Any interface model should be designed towards capturing the physical and chemical processes relevant to the application in question. In this paper we are considering the operation of a solar cell, and in the previous section we have considered the band alignment that is required for the correct distribution of charges in a PV device. In addition to this there are a host of other features required for optimised performance at a PV device interface. We will use a hole-transporting material at the metal-CZTS interface as a motivating example, although the features identified will, in many cases, be transferable to a range of applications and energy harvesting materials and are not solely limited to CZTS. In line with the majority of the literature, we will use CZTS to denote any compound of the form Cu_2_ZnSnS_*x*_Se_4−__*x*_ where *x* can take any value between 0 and 4.

**FIGURE 5:**
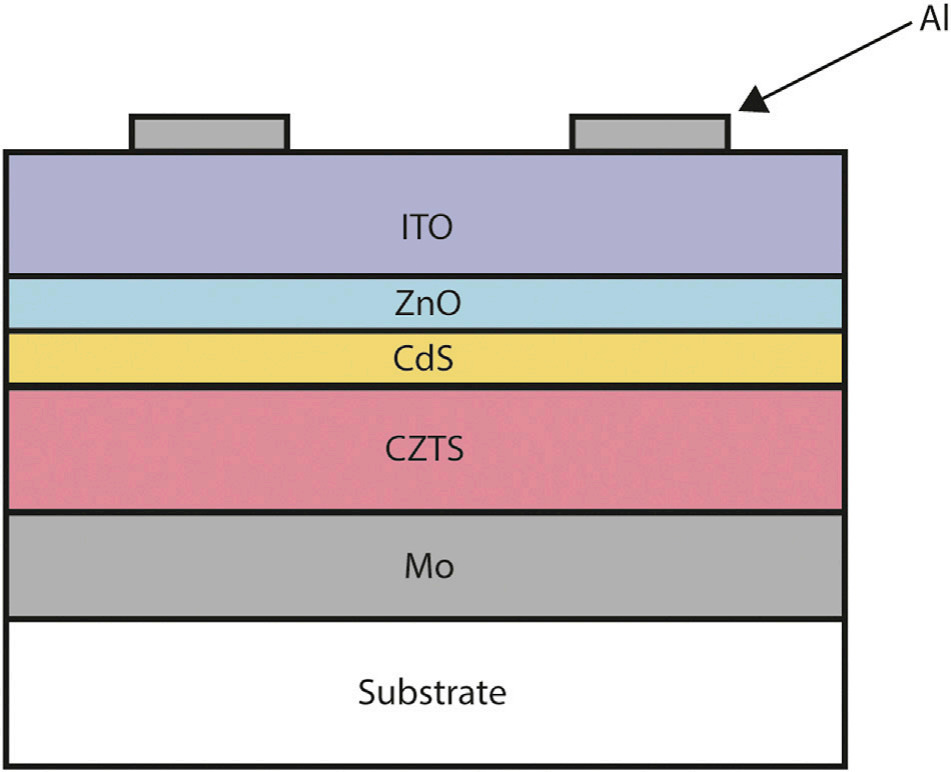
Typical copper zinc tin sulfide (CZTS) solar cell cross-section. From top to bottom: nickel and aluminium front contact grid, indium tin oxide (ITO), intrinsic zinc oxide (ZnO), cadmium sulfate (CdS), copper zinc tin sulfide (CZTS), molybdenum (Mo), soda lime glass substrate. The layer widths are not to scale.

**FIGURE 6:**
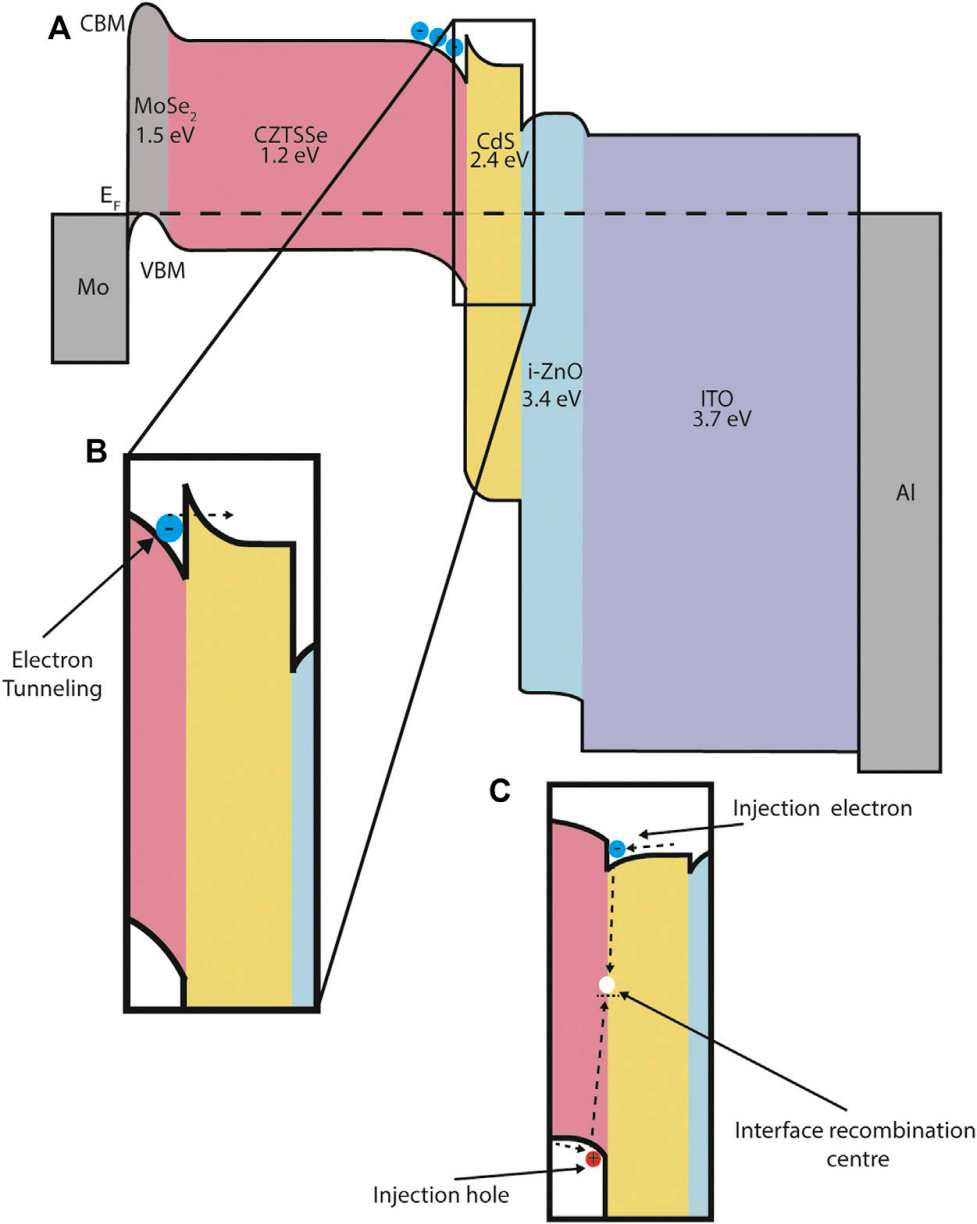
CZTSSe energy band diagram schematic. **(A)** The main panel shows the band alignment across the device. CBM and VBM represent conduction band minimum and valence band maximum respectively. The Fermi-level *E*_*F*_ is denoted by a dashed line. The band gap for each material is given below each chemical formula. All material parameter data is taken from a previously published SCAPS simulation (Campbell et al., 2020). **(B)** Photocurrent resistance at a junction can produce a barrier to electron flow and reduce short circuit current, depending on the energy barrier height. For the CZTSSe/CdS interface a barrier less than 0.4 eV enables transport through a quantum tunnelling mechanism and is beneficial to device performance. **(C)** Alternative cliff-like band alignment at the CZTSSe/CdS interface. In this case injection electrons or holes (the diffusion current) are impeded by the large offset in available energy states and recombine at the interface. This causes a decrease in the reverse saturation current and a reduction in open-circuit voltage and fill factor.

CZTS thin film devices are based on the more established CIGS architecture in which the back contact material is molybdenum metal (Figure 5). As CZTS is a p-type material, the majority carriers are holes and it is these that must be extracted at the back contact. Molybdenum satisfies some qualities of a ‘good’ back contact: it maintains stability at high processing temperatures (Zoppi et al., 2011; Dai et al., 2014) and it has excellent adhesion with the substrate soda lime glass and CZTS absorber layer. However this picture is complicated through the sulfurization/selenisation process used to increase the efficiency of CZTS solar cells. During this process the Mo/CZTS structure is annealed on a substrate in a high temperature tube furnace (Qu et al., 2016a) and a barrier layer of Mo(S,Se)_2_ develops. (Qu et al., 2016b; Xu et al., 2018). The impact of this unintended barrier layer on device performance is still under debate in the literature, with some studies indicating that the formation of Mo(S,Se)_2_ can increase the ohmic nature of the back contact (Figure 6) and produce a contact with lower resistance (Salomé et al., 2010; Wu et al., 2012) providing the thickness of the Mo(S,Se)_2_ is not too large.

### 3.1 Thermodynamic Stability

Thermodynamic stability against decomposition into competing phases is of primary importance, both in the design of bulk and interface materials. The key quantity here is the Gibbs free energy, as this is the value that is minimised for systems in thermodynamic equilibrium. (Jackson and Walsh, 2014). For an accurate understanding of stability the Gibbs free energy for all competing reactions, and at temperatures and pressures relevant for synthesis and device operation, should be considered.

Molybdenum is a commonly used contact material as it can withstand the high temperatures (500–600°C) required for annealing and, as such, provides more flexibility for device design. In contrast, oxides such as MoO_*x*_ and NiO_*x*_ have several decomposition pathways at high temperature and so require deposition in a superstrate configuration after the high temperature anneal.

In addition to establishing the thermal stability of a single material, we must also consider competing reactions at the interface. These reactant and products formed at an interface can incorporate species from each of the contact materials, widening the chemical space to be considered. For example, Scragg *et al.* have described the decomposition of Cu_2_ZnSnS(e)_4_ at high temperatures, both without (Scragg et al., 2012a) (Eq. 4) and with (Scragg et al., 2012b) [Eq. (5)] the presence of molybdenum.

$$2{Cu}_{2}ZnSnS{\left(e\right)}_{4}\to 2{Cu}_{2}S\left(e\right)+2ZnS\left(e\right)+2SnS\left(e\right)+2S\left(e\right)$$ (4)

$$2{Cu}_{2}ZnSnS{\left(e\right)}_{4}+Mo\to 2{Cu}_{2}S\left(e\right)+2ZnS\left(e\right)+2SnS\left(e\right)+MoS\left(e\right)$$ (5)

The free energy change of the reaction with molybdenum [Eq. (5)] is calculated as -100 kJ and -150 kJ at 550°C, for Cu_2_ZnSnSe_4_ and Cu_2_ZnSnS_4_ respectively. These large negative free energies indicate that, at this temperature, the decomposition of CZTS(e) is thermodynamically favourable and that the CZTS(e)/Mo interface is unstable. This demonstrates why molybdenum thin films as a back contact have demonstrated less favourable characteristics, compared to CIGS, when paired with CZTS. It also highlights the importance of the processing techniques used to synthesise high-quality thin-films; in this case high processing temperatures are needed for the improvement of CZTS crystalline structure.

### 3.2 Matched Lattice Parameters

Strain at the interface between two materials is a potential source of instability which can impact on the defect, electrical and optical properties of each material. (Wu et al., 2021). When the strain is below a particular threshold value the material can alleviate the strain through defect formation, which then provide potential sites for charge capture and recombination. Above this value the interface can become incoherent with weak chemical bonding across the boundary. (Butler et al., 2016). The interfacial strain originates primarily from two types of mismatch: mismatch between the lattice constant of each material, and mismatch between the thermal expansion coefficient (*α*) of each material.

Lattice mismatch and strain related defects have been of particular importance in the development of high-efficiency III-V multijunction solar cells. With conversion efficiencies now approaching 50%, these cells are fabricated using epitaxial growth and careful control of the atomic-scale structure. (Li et al., 2021). A pre-requisite for good performance is that each absorber layer is lattice-matched to its neighbours, which is the predominant reason that the absorber layers in the highest-efficiency multijunction devices consist of closely related materials from the III-V compounds, such as GaAs, GaInP and GaInAs.

Various models have been introduced to rationalise the strain formed at the boundary between two materials and to identify alternative contact materials. For materials with the same crystal structure (for example III-V compounds in the zincblende structure) a comparison of the lattice parameters will suffice, whilst for materials with different crystal structures the comparison requires more subtlety. In 1984 Zur and McGill developed a reduction scheme based on the primitive lattice vectors of each material, (Zur and McGill, 1984), which has been more recently applied to a screening study for hybrid perovskite contact layers, (Butler et al., 2016), and implemented in the ElectronicLatticeMatch code. (Butler, 2017). To account for chemical bonding, which is neglected in the Zur and McGill scheme, more advanced theories that account for the atomic species on each lattice site are required. (Park et al., 2018).

Matched thermal expansion coefficients are also important due to the high annealing temperatures often used to enhance crystallinity and reduce defects in thin film PV; for example, CsPbI_3_ and CZTS are typically annealed at temperatures up to 330°C and 600°C respectively. These high temperatures result in a large temperature gradient during cooling to ambient temperatures. If combined with highly mismatched thermal expansion coefficients, a large thermally-induced strain results. (Wu et al., 2021).

In contrast to the lattice matching techniques introduced above, recent research has also harnessed the lattice mismatch between a material and substrate. This mismatch is used to facilitate a lift-off process, where the active material(s) are synthesised on a rigid substrate and then removed using physical, chemical or thermal methods. (Liu et al., 2021). For thermal lift-off processes the substrate is selected to have a different thermal expansion coefficient. When cooling a large stress field develops which is alleviated by the development of cracks parallel to the surface, and which eventually results in delamination of the active material. (Dross et al., 2007).

### 3.3 Energy Band Alignment

As already discussed, energy band alignment is also of critical importance, as this determines the rate of charge transport across an interface. At a metal-semiconductor junction the key quantity is the Schottky barrier height which relates to the difference in energy between the metal and semiconductor work functions. A contact is considered ohmic when the Schottky barrier height is non-existent or small enough to allow for unhindered transport of the electrons (for n-type materials) or holes (for p-type materials) across the interface.

At the CZTS back contact, there is evidence that the formation of a MoS(e)_2_ layer creates an ohmic contact with the CZTS layer. However the overrall PV performance may be reduced due to increased recombination of electron and hole charge carriers at the Mo/MoS(e)_2_ interface. The former observation is in line with research for closely related CIGS-based technologies, where it has also been suggested that MoS_2_ improves the ohmic nature of the Mo/CIGS back contact. (Wada et al., 2001; Polizzotti et al., 2013). However if the MoS_2_ layer is too thick this will be detrimental to device performance, resulting in a reduction in V_OC_ and an increase in the series resistance. (Shin et al., 2012; Xu et al., 2019).

For CZTS an additional complication is the trade-off between thermodynamic stability of CZTS and the thickness of the MoS(e)_2_ layer. To prevent the degradation of CZTS through the loss of 2S(e) during high-temperature annealing, over-pressures of S(e) are used. However these high gas pressures cause the formation of a thicker layer of MoS(e)_2_ as a by-product.

Another important interface in CIGS and CZTS devices is the interface with the buffer layer, most commonly CdS. (Courel et al., 2014; Courel et al., 2015). There are two main types of conduction band offset (CBO) that occur at this interface: a positive, spike-like band alignment, where the buffer layer CB is higher than that of the absorber layer, and a negative, cliff-like, where the buffer layer conduction band (CB) is lower than that of the absorber layer (Figure 6).

In CIGS the most optimum CBO at the CIGS/CdS interface is, counter-intuitively, spike-like rather than cliff-like, providing that the barrier height is below 0.4 eV. This barrier allows electrons to tunnel through into the CdS, but does not produce a barrier to injection electrons under forward bias. The quantum tunnelling mechanism is described in more detail in Section 6.1. For cliff-like junctions, accumulated injection electrons under forward bias result in an increased recombination mechanism at the interface and a lower V_OC_. A spike-like energy barrier greater than 0.4 eV forms a significant barrier to current flow resulting in a dramatic decrease of the short-circuit current. (Minemoto et al., 2001; Campbell et al., 2020).

### 3.4 Electrically Benign Defect Formation

The formation of material defects, whether in the bulk or at interfaces, cannot be avoided in most materials. (Walsh and Zunger, 2017).

Furthermore, these defects can have serious unwanted implications in a PV device. Of particular concern are electrically active defect states that can capture electrons and/or holes, as these can lead to a reduction in the open-circuit voltage (V_OC_) and fill factor (FF), through processes such as Fermi-level pinning or non-radiative recombination.

The band bending model introduced in Section 2 effectively breaks down when we start to introduce material imperfections at the semiconductor-metal interface (Figure 6C). There are two possibilities that can arise at a metal-semiconductor interface: metal-induced states and semiconductor-vacuum states. In both instances electrically-active defect levels can be created within the semiconductor band gap, but each are associated with a different mechanism. In the former the states are created upon contact with the metal and are induced, for example, by lattice strain or chemical bonding. The latter are present on the surface of the semiconductor interface without contact to other materials.

Defect states in the band gap can cause a phenomenon known as Fermi-level pinning. Fermi-level pinning occurs when the interface defect states trap charge carriers that diffuse from the metal into the semiconductor. This effectively isolates the semiconductor from the effects of the metal and the semiconductor bands are aligned (pinned) relative to the charge neutrality level (CNL) of the interface defect states. This process adjusts the potential and electrostatic field at the interface, which in turn adjusts carrier transport across the interface. (Dimoulas et al., 2006).

In Ge-metal contacts Fermi-level pinning is a strong limiting factor in depositing contact grids with ideal electrical behaviour. The charge neutrality level (CNL) in Ge is situated close to the valence band (0.09 eV above *E*_*V*_), so that most acceptor states at the interface are filled at ambient temperature. (Dimoulas et al., 2006). As such, the Schottky barrier height that forms at this interface shows weak dependence on the particular metal used for contact to the electrical circuit. This is confirmed by a later study revealing that metal/*p*-Ge and metal/*n*-Ge junctions have ohmic and Schottky characteristics respectively, and both display a strong degree of Fermi-level pinning. (Nishimura et al., 2007).

Defects at the CZTS/CdS and CZTS/MoS(e)_2_ interfaces are abundant due to the low formation energies required for exchanging ion species of the same type (in this case, sulfur or selenium) across an interface. In addition, Cu and Zn have comparable ionic radii resulting in low formation energies for Cu_*Zn*_ and Zn_*Cu*_ antisite defects in CZTS. This is compounded by the high annealing temperatures required for the growth of high-quality CZTS grains. (Chen et al., 2013). A large accumulation of antisite defects at the interface results in insufficient type inversion (the majority carrier being the opposite of its host layer) and weak band bending as an effect of Fermi-level pinning, whereby the Fermi-level is pinned close to the valence band at the interface. (Yuan et al., 2015). Cu-Zn disorder is also associated with band-tailing, where the measured band gap is less than that expected for the perfect bulk material. This fluctuation is dependent on the chemical purity of the precursor used to synthesise CZTS, and so the density of defects that are expected to be present. (Campbell et al., 2019).

Most studies of interfacial defects in CZTS solar cell devices focus on the CZTS/CdS or CZTS/MoS(e)_2_/Mo interfaces. (Kaur et al., 2017; Qi et al., 2017; Suryawanshi et al., 2017; Campbell et al., 2018; Kim J et al., 2020). Defects that are particularly detrimental to performance are observed at the CZTS/CdS interface, where a high concentration of defects results in cliff-like band alignment and high non-radiative electron-hole recombination rates. This is also observed at the CIGS/CdS interface. (Kaur et al., 2017).

In addition to defect formation at semiconductor-semiconductor and semiconductor-metal heterojunctions, CZTS, CIGS and halide perovskite thin films are particularly prone to the development of grain boundary (GB) defects. (Yan et al., 2007; Li et al., 2011; Park et al., 2019). Not all GB defect formation is problematic; for example there is evidence that GBs in CIS-based materials are beneficial to device performance. (Yan et al., 2007). In contrast, the defect states associated with GBs in CZTSe are located in the band gap and as such provide sites for charge trapping and recombination. (Li et al., 2011). Similarly, the GBs in halide perovskite materials are detrimental to device performance in that they provide energetically favourable sites for the formation of iodine interstitial defects, which in turn act as a site for charge trapping and recombination. (Park et al., 2019).

### 3.5 Favourable Ion Transport

Ion transport, when compared to electronic transport, is not as central to the functioning of a solar cell. However it can still influence the performance of a device through material doping and charge accumulation. For some materials, such as CZTS and CIGS, ion diffusion can increase the cell performance. In others, such as the halide perovskites, ion migration can lead to decreased performance. Examples for both extremes will be given in this section.

CZTS based solar cells are often fabricated on a soda lime glass (SLG) substrate, which primarily provides mechanical support to the cell. This results in sodium from the SLG also diffusing through the device into the MoSe_2_, Mo and CZTS layers, leading to a notable improvement in the conductivity of the CZTS layer. (Prabhakar and Jampana, 2011). In a study comparing SLG to other substrates, the carrier concentration in CZTS increases by an order of magnitude (6.1 × 10^16^ to 35.4 × 10^16^ *cm* ^−3^), leading to a significant increase in conductivity (from 41.2 to 58.4 Ω*cm* ^−1^). (Prabhakar and Jampana, 2011). The underlying reasons for this increased carrier concentration has been examined using first-principles calculations. Wei and Zunger determined that sodium on a copper site (Na_*Cu*_) results in an injection of holes into the system, increasing the hole density and thus the conduction of the CZTS p-type absorber layer. Sodium intercalation has also been associated with an increase in crystallite size, reducing the grain boundary area which in turn reduces the number of locations for non-radiative carrier recombination. (Prabhakar and Jampana, 2011). This same relationship has been observed in CIGS films fabricated on soda lime glass substrates. (Contreras et al., 1998).

Another source of beneficial ions in a CZTS cell is at the molybdenum rear contact. During the sulfurisation/selenisation process molybdenum is exposed to temperatures between 500–600°C, at which the molybdenum crystal structure should be relatively unchanged. However there is a clear temperature-dependent diffusion of molybdenum into the CZTS layer, with sulfurisation at 600°C leading to a more Mo-rich CZTS interfacial layer and PV efficiencies five times greater than materials formed *via* sulfurisation at 500°C. These studies demonstrate the importance of contact materials being able to maintain a stable crystal structure whilst still allowing beneficial ion diffusion throughout the device.

On the other hand, ion diffusion in perovskite-based PV negatively affects stability, performance, and effective working lifetime. Hybrid halide perovskites (ABX_3_) are categorised as mixed conductors meaning they possess both electronic (Leijtens et al., 2015a) and ionic conductivity, (Eames et al., 2015; Egger et al., 2015; Yang et al., 2015), a phenomenon which has been extensively studied in recent years. (Xiao et al., 2015; Xu et al., 2015; Thomas et al., 2016). Ion transport in these materials is the primary contributing mechanism for I-V hysteresis (Meloni et al., 2016) and poor thermal stability, (Leijtens et al., 2015b; Zhao et al., 2016), leading to performance degradation over time. (Leijtens et al., 2015c). These materials also display a coupling between electrical and ionic behaviours, with increasing light intensity causing a reduction in the activation energy for ion migration. (Zhao et al., 2017).

In this section we have outlined several properties and processes at the interface that impact on device performance. We note here that this is a challenge not only for materials design, but also for materials process engineering. For example, to reduce the concentration of electrically active point defects various strategies have been developed: selecting high-quality precursors for materials synthesis, (Campbell et al., 2019), incorporating additional dielectric passivation layers, (Kim et al., 2017), or using post-deposition annealing. (Fritsche et al., 2005; Jaegermann et al., 2009).

We also note that our ‘wish-list’ for an ideal PV material is somewhat incomplete and could be extended to include other factors we do not mention here. This includes thermal transport properties (the rate of heat transfer), mechanical properties (elastic modulus and deformation) and optical properties (band gap and absorption coefficients). On the prior point we draw the readers attention briefly to Figure 6, which shows a series semiconductor materials with increasing band gap as we move between the back metal contact (Mo) and the front metal contact (Al). Increased optical band gaps result in increased ultraviolet light transmission and more light energy available for conversion within the absorber material.

In the next three sections we review methods for modelling the interface between two materials. We begin with a short section on chemical heuristics—using simple chemical rules to reduce the vast compositional space of possible interface materials. We then review atomistic methods for understanding at an atomistic level the complex physical and chemical processes at an interface. Finally, we outline the theory and techniques for modelling interfaces at the device level.

## 4 Data-Driven Approaches to Interface Materials Modelling

A common approach when developing a photvoltaic absorber material is to integrate the new material into the existing device architecture for a related compound. For example, CZTS in the kesterite structure is derived from CIGS in the chalcopyrite structure. As a result of this relation, CZTS thin film devices are based on the CIGS architecture with a CdS window layer and molybdenum back contact. However this transfer between technologies is unlikely to lead to a optimal material pairing because, as we have discussed above, processes at an interface are highly sensitive to the specific structural, electronic and defect properties of a material.

The opposite data-driven approach is to widen the search space to include all possible interface materials, and then apply a sequence of computational filters to identify those with a set of pre-defined target properties. The initial filters may include low-cost chemical rules (also known as chemical heuristics) to reduce the search space. For example, if searching for a binary material we might enforce the first Pauling rule, requiring that the ratio of the anion and cation ionic radii falls within a certain range. (Pauling, 1929). Alternatively, the filters may not derive from chemical knowledge but from statistical (e.g machine learning) models built on a large dataset of material properties. An advantage of the screening approach is that additional constraints can be implemented in the workflow. For example, the Herfindahl Hirschman index can be applied to select for compounds which contain abundantly available elements, (Mansouri Tehrani et al., 2017), or compounds containing toxic elements can be removed at an early stage.

When an atomic-scale structure is yet to be determined the computational model must be able to make predictions based on elemental composition only. In the context of interfaces in solar cell devices we are particularly interested in predicting the electronic properties of materials, such as the CBM and VBM referenced to the vacuum level, and the electronic band gap. Chemical rules connecting elemental composition with electronic structure have a long history starting with the work of Mulliken who developed an absolute scale of atomic electronegativity (defined as the mid-point in a semiconductor band gap) in 1934. More recently, Pelatt *et al.* proposed the Atomic Solid State Energy (SSE) scale as an alternative approach to electronegativity. (Pelatt et al., 2011). This is derived from data for the ionisation potentials and electron affinities of 69 binary semiconductors containing 40 different elements. By considering the energy difference between the most negative cation SSE and the least negative anion SSE, this method provides estimates for the absolute CBM, VBM and band gap in any compound. The SSE scale has been used as a filter in a high-throughput search for photoactive chalcohalide semiconductors, identifying two new compounds with band gaps in the visible spectrum, (Davies et al., 2018), and to rationalise the measured hole concentrations in Cu-based chalcogenide PV absorber materials. (Itthibenchapong et al., 2013).

The SSE scale is based on a linear trend between the electron affinity and ionisation potential versus band gap. However for many properties of interest the trend is not so readily recognisable as there may be a higher number of dependent parameters and/or more complex non-linear relationships. In this case machine learning (ML) can be used to identify relationships in the data and develop predictive models.

The number of studies based on ML applied to materials science is growing quickly. This is driven by investment into high-performance computing, freely available materials databases [for example the Materials Project (Jain et al., 2013) or OQMD (Kirklin et al., 2015)] and open-source machine learning libraries. A common approach is to use well-known chemical concepts, such as ionic radii or electronegativity of the constituent elements, as features for supervised machine learning. This approach has been used in a number of studies to predict the electronic band gap across a range of potential materials for solar applications, including: double chalcogenide perovskites, (Agiorgousis et al., 2019), orthorhombic lead-free perovskites, (Lu et al., 2018), wurtzite nitride semiconductors (Huang et al., 2019) and kesterite materials. (Weston and Stampfl, 2018). The predictive power of the machine learning model depends on there being a training database of suitable size, hence the reliance on either existing materials data or high-throughput computing resources.

ML has also been used in the development of interatomic potentials. Here the ML model is trained to predict energies and forces from first-principles calculations of small systems (tens or hundreds of atoms). The ML-based potential (MLP) is then used to model large systems (thousands of atoms) that are intractable for first-principles simulation. This approach has particular relevance to interface materials where amorphous and nano-structured phases can be formed which require large periodic unit cells for building accurate models. For example, a MLP for molybdenum has been developed that achieves close to DFT accuracy for a broad range of properties including elastic constants, phonon spectra and surface energies. (Chen et al., 2017). This MLP is based on the spectral neighbor analysis potential (SNAP) developed by (Thompson et al., 2016) It also incorporates well-established, domain-agnostic machine learning techniques: principal component analysis for the structural selection process, and a differential evolution algorithm for optimizing the model hyperparameters.

The studies introduced so far in this section have been used to predict the properties of a single material. There are also a small number of studies that use ML to predict the chemical and structural changes that occur when two materials join to form an interface. For example, ML has been used to predict the structures formed at epitaxial inorganic interfaces typical of high-efficiency III-V PV materials. This technique uses surface matching accelerated with Bayesian optimisation to predict the interfacial distance and potential energy surface at a fraction of the computational cost required by the equivalent DFT calculations. (Moayedpour et al., 2021). It is also possible to use a MLP to describe interface bonding if a suitable training set is used. There are various cases in which this approach has been used to describe systems where the interface region is small, such as in supported nanoclusters. (Artrith, 2019). Modelling the interface between two extended crystalline materials is more challenging as this typically requires larger simulation cells to generate the training data. The grain boundaries in aluminium have been modelled using a MLP which was trained using structures optimised using conventional interatomic potentials and density functional theory. (Tamura et al., 2017). This allowed the prediction of minimum energy structures for extended grain boundary structures that would not have been accessible using DFT alone. There is not yet, to our knowledge, extension of this approach to other materials or heterogeneous interfaces.

The on-going challenge for materials simulation is the trade-off between accuracy and computer time. The common theme across all of the ML-based studies introduced in this section is that they aim to provide predictions with the accuracy of ab-initio quantum chemical simulations but at lower cost. Although this computational efficiency has been established for accurately predicting the properties of a single material, there has been minimal data-driven research which explicitly considers the bonding and structural changes at a PV interface. For these research problems the most commonly used approach is classical or quantum atomistic simulation, which is the subject of the following section.

## 5 Atomistic Modelling of Photovoltaic Interfaces

Given that materials interfaces represent major bottlenecks to the performance and stability of PV devices, their accurate simulation and understanding at the atomistic scale are becoming ever more important. However, compared to the modelling of bulk crystalline materials, the methods for modelling interfaces in PV materials (and indeed other energy materials) are far less developed and often do not present the same reliability and computational efficiency. While bulk crystalline materials are modelled atomistically as infinite lattices using three-dimensional periodic boundary conditions, simulations of the heterogenous interfaces in PV devices require the preparation of surface slabs that are placed in direct contact with each other. The development of such interfacial models is challenging and careful consideration must be given to their scale, alignment, chemical composition, stability, disorder/defects and thermal and mechanical damage to ensure that they are reliable and representative of real interfaces. Nevertheless, despite the challenges they present, the atomistic modelling of interfaces can offer a wealth of valuable information regarding the performance of PV materials and devices at an atomic resolution, as well as the potential for the discovery of unique behaviour and properties not exhibited in bulk materials.

Thermodynamic stability is one of the most fundamental properties for defining the pertinency of a given interface. The first factors to consider when creating an interfacial model are surface formation and the energy it requires, i.e., the surface free energy (*γ*_surface_, J m^-2^). *γ*_surface_ defines the energy required to cleave a bulk material and form a surface with a particular Miller index and can be calculated using both forcefield or electronic structure methods, as exemplified in many atomistic modelling studies of PV materials. (Raymand et al., 2008; Wang et al., 2015; Nicholson et al., 2020). Such a surface can then be modelled as a slab consisting of a number of atomic layers converged with respect to the calculated internal energies from DFT or a forcefield approach. Several approaches have been proposed to reduce the computational expense of converging the slab thicknesses, including orientating the reference bulk material with the Miller index of the surface of interest (Sun and Ceder, 2013) and saturating any dangling bonds with pseudo-hydrogens based on fractional core and electronic charges. (Sai et al., 2018). By combining the *γ*_surface_ values for a selection of Miller index planes, the Wulff construction, which minimises the surface energy for a given enclosed volume, can be used to predict the equilibrium particle morphologies of PV materials. (Barnard and Curtiss, 2005; Wilson et al., 2016).

The energy of an interface (*γ*_interface_) can also be obtained using a similar method to that for *γ*_surface_ using the computed total energies of the interface and its corresponding bulk materials. Like *γ*_surface_, *γ*_interface_ must also be converged with regards to system size, which can represent a substantial computational cost, particularly for quantum mechanical simulations. The sign of *γ*_interface_ signifies whether the interface is more (negative value) or less (positive value) stable than the respective bulk materials in isolation. This method has been used to provide significant insights into the stability of the interfaces of PV materials, for example CZTS/CdS (Rondiya et al., 2019; Rondiya et al., 2020), CCTS/CdS (Rondiya et al., 2020) and TiO_2_/hybrid perovskites. (Mosconi et al., 2014). In addition to heterogenous interfaces, *γ*_interface_ is equally applicable to homogenous interfaces and has been widely applied to investigate the stability and formation of grain boundaries in various PV materials. (Park and Walsh, 2021).

As discussed earlier in the review, the alignment of bands between different materials is critical to the performance of devices with semiconductor heterojunctions. Quantum mechanical simulations play an important role in predicting and optimising band alignment and the most used approach to provide a comparison with experiment is the use of slab models. (Singh-Miller and Marzari, 2009; Peng et al., 2013). In this approach, the electrostatic potential in the vacuum provides a reference so that the ionisation potential can be calculated using the valence band maximum of the slab model. One issue that can arise from the use of slab models based on ideal surface cuts in calculating the ionisation potential is the presence of dangling bonds. Although these surface states mean that the ionisation potential from a single slab does not represent the true ionisation potential from the bulk material, their effect can be corrected for by using the calculated valence band maximum of the bulk material and surface dipole of the slab. (Peressi et al., 1998). It is also important to bear in mind that the use of idealised surface models does not take into account the effect of impurities (either added intentionally or otherwise), which can have a dramatic effect on the electronic properties of the interface. (Davis et al., 1980; Ming et al., 2018; Borchert et al., 2019).

In addition to the use of explicit interfacial models, two alternative approaches have been utilised for the calculation of band alignment *via* atomistic modelling. The first is the natural band alignment approach, where the band offsets are obtained by explicitly calculating the heterojunctions of the semiconductors of interest. While this approach has the advantage of allowing for facile comparison with spectroscopic analyses, it is only appropriate for materials with similar structures and compositions due to the assumption of transitivity for the band offsets. (Butler et al., 2019).

This method has been successfully applied to investigate band alignment in a wide variety of PV materials, including II–VI and III–V semiconductors (Wei and Zunger, 1998) and hybrid perovskites. (Butler et al., 2015). The second technique utilises the branch-point energy, which is defined as a level at which defect states in the band gap change from donor-like to acceptor-like states, to align bands across bulk semiconductors. This can be achieved by calculating the band gap centre, which represents the average of the VBM and CBM across the Brillouin zone (Toroker et al., 2011). Although this approach is relatively straightforward, computationally inexpensive and has been applied to a large selection of oxide and nitride PV materials, (Schleife et al., 2009), it is highly sensitive to the choice of computational parameters. (Alidoust et al., 2014). In-depth reviews of these methods and their application to PV interfaces are available elsewhere. (De Angelis, 2014; Even et al., 2014; Park and Walsh, 2021).

It is also noteworthy that atomistic modelling can also potentially be used to assess a variety of other important properties that govern the performance of PV interfaces beyond thermodynamic stability, electronic structure and band alignment, such as ion transport, defects and degradation mechanisms. However, despite the importance of these properties and the fact they have been routinely simulated in individual PV materials (Oba et al., 2008; Eames et al., 2015; Lanzetta et al., 2021) and grain boundaries (Park and Walsh, 2021) using both classical and quantum methods, studies of their effect on PV heterointerfaces are at best scarce. This is primarily because of the inherent challenges in modelling interfaces discussed throughout this review.

Due to their own unique advantages and disadvantages, the application of both forcefield and electronic structure calculations is vital in closing the current gap between bulk and interfacial modelling in terms of practicality, efficiency and accuracy. The need for larger atomistic models that can be simulated for longer timescales is one of the most critical challenges currently facing the modelling of interfaces and this is where classical techniques, such as molecular dynamics, have a distinct advantage. Such models can also be parameterised using DFT and ML calculations to improve their accuracy and reliability. In contrast, there are clearly many properties, e.g., band alignment and charge transfer, where the electronic structure of the interface must be considered explicitly and the use of classical forcefields is not appropriate. The understanding of the interfaces in PV devices is inherently a multi-scale problem and therefore require multi-scale approaches to solve it.

## 6 Continuum Scale Models of Photovoltaic Devices

The simulation of a complete solar energy conversion system is a macro-scale problem that requires us to consider several material interfaces. In previous sections we have discussed modelling material structure and composition both in a bulk interface material, and at the interface itself. Here we consider models at the device level, which can incorporate several material interfaces, and include simulations up to the scale of module installations.

There are several approaches for calculating the electronic properties of a device. At the simplest level there is the detailed balance approach, which in some formalisms only requires the energy and absorptivity of the absorption edge. For increased accuracy, but where a detailed knowledge of the device structure is unknown, a diode model can be used. In this case, the various transport and recombination mechanisms can be modelled using electrical components. However the most fundamental and accurate method for predicting electronic behaviour is Poisson-drift-diffusion (PDD). This underlies the large majority of software tools used for modelling PV devices, and is the focus of the following section. Following this we summarise and discuss some of the key software packages used for device modelling and identify opportunities for accelerating our ability to model interfaces in a increasingly diverse array of PV devices.

### 6.1 Equations Governing Device Level Behaviour

The most fundamental equation for predicting electronic behaviour in semiconductor devices is the Poisson equation, which relates the electrical charges in the structure to the electrostatic potential *ϕ*. In a semiconductor the charge is typically split onto four density domains: electron density *n*, hole density *p*, acceptor atom density $N_{A}^{-}$ and donor atom density ${N_{D}}^{+}$ . The Poisson equation in one dimension is given by:

$$\frac{d}{dx}\left(\epsilon \frac{d\phi }{dx}\right)=-q\left(p\left(x\right)-n\left(x\right)-N_{\text{A}}^{-}+N_{\text{D}}^{+}\right),$$ (6)

where *ϵ* is the dielectric permittivity of the material. The continuity equations are used in conjunction with Poisson statistics. The continuity equations are book-keeping equations in that they ensure conservation of charge, balancing the carriers that enter and leave different parts of the model. The continuity equations for free electrons in the conduction band and holes in the valence band are given by the following two equations:

$$\frac{1}{q}\frac{dJ_{n}}{dx}=U\left(x\right)-G\left(x\right)$$ (7)

$$\frac{1}{q}\frac{dJ_{p}}{dx}=-U\left(x\right)+G\left.\left(x\right)\right),$$ (8)

where *J*_*n*_ is the electron current density, *J*_*p*_ is the hole current density, *G*(*x*) is the generation rate (for a solar cell this will be generation from illumination) and *U*(*x*) is the net recombination rate. The recombination rate may incorporate several mechanisms including radiative band-to-band recombination, defect mediated Shockley-Reed-Hall recombination, Auger recombination and surface recombination at the interface between materials. The equations describe a steady-state system where the change in current is exactly balanced by charge generation and recombination.

The transport equations describe the dynamics of the carriers and therefore also the current in a solar cell. Current in a solar cell can be broken down into two parts: a drift component due to the electric field $E=-\frac{d\phi }{dx}$ and a diffusion component due to the carrier concentration gradient. This can be seen in the transport equations, where the first term correlates to drift current and the second term to diffusion current:

$$\frac{1}{q}J_{n}=-\mu_{n}n\frac{d\phi }{dx}+D_{n}\frac{dn}{dx}$$ (9)

$$\frac{1}{q}J_{p}=-\mu_{p}p\frac{d\phi }{dx}+D_{p}\frac{dp}{dx},$$ (10)

where the as-yet undefined constants are material-specific transport parameters: *μ*_*n*_ is the electron mobility, *μ*_*p*_ is the hole mobility, *D*_*n*_ is the electron diffusivity and *D*_*p*_ is the hole diffusivity.

The above equations are well-established equations for semiconductors that are derived and expanded on in many semiconductor textbooks [for example, Reference (Nelson, 2003)]. They form a coupled system of nonlinear partial differential equations which do not, in general, admit analytical solutions for systems of interest. Instead, numerical techniques are used to model the behaviour or these systems for given material types and geometries. These numerical techniques are all based on the discretisation of a device in both space and time yet can be approached in a variety of ways, such as the well-established and robust finite difference method, or the finite element method which is more flexible in terms of problem geometry. In addition, there are various alogrithms which implement each method. For example, the finite element method can be implemented using the Gummel method for fast convergence, or the Newton method when there are high recombination rates leading to strong coupling of the continuity and Poisson equations. (Liu et al., 2011). Once solved, the equations describe the movement of charge carriers and their relationship with current and electric field strength.

In addition to the classical physics introduced above, quantum tunnelling should be considered for the accurate prediction of charge transport behaviour across heterojunctions where either the material layer or depletion layer is thin (typically less than 15 nm). Quantum tunnelling can affect the calculated current as it results in the transport of electrons across a potential barrier even if the electron energy is less than the barrier height—transport that is forbidden in the classical regime. Experimental measurements demonstrate that quantum tunnelling mechanisms can dominate the charge transport current behaviour in a cell and can significantly impact the PV performance when there is a spike-like barrier at the interface. (Verschraegen and Burgelman, 2007). For a full description of quantum tunnelling, electrons with an energy below the potential barrier can be represented by the Schrödinger equation and solved to give wave vectors and a tunnelling probability. This however requires computational resources beyond that typically used for device level simulations, and so analytical approaches such as the WKB method are commonly used to give approximate corrections to the calculated current. (Burstein and Lundqvist, 1969; Gundlach and Simmons, 1969; Verschraegen and Burgelman, 2007).

There are also multi-scale device models that incorporate methods for solving the Schrödinger equation directly. These models are motivated by the increasing amount of research and development into solar cell devices based on nano-structured materials, such as quantum dot, nanowire or quantum well structures. These materials consist of multiple nanometre thin layers which can, as a result of being ultra-thin, accommodate large amount of strain at the interface. (Kroemer, 2001). Research interest is driven by their potential for increased conversion efficiencies over single-junction cells without the complexity of multi-junction designs. In several software packages (Birner et al., 2007; Alonso-Álvarez et al., 2018) the quantum properties of the nanostructures are calculated by solving the one-dimensional Schrödinger equation to give the eigenvalues and eigenvectors associated a given potential. To include local strain fields that develop as a result of lattice deformation the electron mass can be modified. (Birner et al., 2007; Alonso-Álvarez et al., 2018). It is also possible to repeatedly iterate through the Schrodinger and Poisson equations until a self-consistent solution that takes into account the classical and quantum mechanical charge densities has been found. (Birner et al., 2007).

As discussed further in the following section, these classical and quantum techniques are implemented in several software packages which are designed to output useful semiconductor characterisation data that can be directly compared to experimental results. Notable examples relevant for PV research include current-voltage (J-V) characteristics, internal quantum efficiency (IQE) and external quantum efficiency (EQE) and there are also software packages that can make predictions for more advanced characterisation techniques such as capacity-voltage and photoluminescence. (Froitzheim et al., 2003).

### 6.2 Solar Cell Modelling Packages

There are numerous packages to simulate crystalline, polycrystalline and nano-engineered PV cells. Many of these are well documented and have yielded significant insights whether paired with experimental results (Karthick et al., 2020; Houimi et al., 2021) or as stand-alone simulations. (Verschraegen and Burgelman, 2007; Feng et al., 2013; Mostefaoui et al., 2015). Whilst these packages tend to simulate the same sets of solar cell characterisation data (for example, current-voltage curves) they are differentiated by both the approach they use and the applications they are tailored towards. For example, nextnano (Birner et al., 2007) uses the finite element method and is particularly suited to the diverse geometries of nano-engineered materials. In contrast, PC3D uses a fourier transform solution to the drift-diffusion equations which is computationally efficient but more restricted in its applications. (Basore, 2020).

Table 1 summarises some of the other notable software packages for PV device simulation. The packages listed are capable of exploring several aspects of solar cell device behaviour, with the primary focus being on semiconductor physics. In addition to those listed in the table there are packages tailored towards simulating other sub-sets of the PV energy generation process, including SMARTS to calculate the solar spectrum as a function of atmospheric conditions, (Gueymard, 1995), OPTOS for light absorption at the module level (Tucher et al., 2016) and PVlib for module and systems level modelling. (Holmgren et al., 2018a).

**TABLE 1:** A non-exhaustive list of solar cell device simulation tools. The table allows a comparison of the key features and availability. Unless otherwise stated, the simulations are one-dimensional and a graphical user interface is available. ‘Open source’ indicates that the source code is available for free download. ‘Freely available’ indicates that the compiled software is available for free download. Note that Solcore is free to use and is distributed with an open source license, GNU LGPL (gnu.org/licenses/lgpl-3.0). We also include signposts for further information: a project web address and a reference in the academic literature. Finally we list selected publications in which the software has been applied.

Name	Features	Availability	Web Address	Reference	Applications
SCAPS	• widely used in academica • intra-band, band-to-band and interface defect tunnelling implemented	freely available	scaps.elis.ugent.be	Burgelman et al. (2000)	Verschraegen and Burgelman, (2007), Karthick et al. (2020), Houimi et al. (2021), Mostefaoui et al. (2015), Simya et al. (2015), AlZoubi and Moustafa, (2019), Jannat et al. (2021)
Solcore	• modular and extendable • no graphical user interface • Schrodinger solver for quantum mechanical properties	GNU LGPL	solcore.solar	Alonso-Álvarez et al. (2018)	Führer et al. (2013), Nikander et al. (2020)
PC3D	• for silicon solar cells only • simulations in 3D • Excel-based user interface	open source	pc3d.info	Basore, (2020), Basore, (2018)	Balaji et al. (2020)
wxAmps	• based on the AMPS code • Newton and Gummel methods for faster convergence	open source	github.com/wxAMPS	Liu et al. (2012a) and Liu et al. (2012b)	Yaşar et al. (2016), Chen et al. (2016)
Victory Device	• general purpose device simulator • simulations in 2D and 3D • electrical, optical and thermal properties	paid license	silvaco.com	Michael et al. (2005), Michael, (2005)	Elbar and Tobbeche, (2015)
Sentaurus	• general purpose device simulator • simulations in 2D and 3D • electrical, optical and thermal properties	paid license	synopsys.com	Wu and Jhan, (2018)	Passeri et al. (2016), Limpert et al. (2014)
Quokka3	• optimised for silicon cells • simulations in 1D, 2D and 3D	free and paid licenses	quokka3.com	Fell, (2012)	Fell and Altermatt, (2018), Richter et al. (2017)
AFORS-HET	• includes advanced characterisation techniques such as capacity-voltage and photoluminescence	freely available	helmholtz-berlin.de	Froitzheim et al. (2003), Rolf et al. (2006)	Yao et al. (2018), Wang et al. (2011)
nextnano	• optoelectronic device simulator • Schrodinger solver for quantum mechanical properties	paid software	nextnano.de	Birner et al. (2007)	Refaei, (2017)

The availability of each code is also listed in Table 1. The general purpose device simulators, capable of modelling a whole host of semiconductor devices including memory devices and power electronics, are proprietary and require a paid license. Of those listed that are freely available, only Solcore is distributed with an open source license (GNU LGPL, gnu. org/licenses/lgpl-3.0) although the source code for PC3D and wxAmps is openly available. Two of the software packages listed, Quokka3 and PC3D, are heavily geared towards silicon devices, and are the only freely available packages listed for simulations in three dimensions. The user interfaces come in a variety of forms: Solcore provides a library of tools which are called directly in a Python script, PC3D uses an excel-based user interface, whilst others (including SCAPS, wxAmps and AFORS-HET) provide a graphical user interface.

The list of simulation codes in Table 1 is not exhaustive. Several groups develop their own in-house codes tailored towards particular research problems, some of which have been developed and published in public code repositories. (Holmgren et al., 2018b; Juan, 2022; Koopmans and Vincent, 2022). These codes generally provide command line interface (CLI) or application programming interface (API) to underlying routines, and as such require a basic familiarity with programming. Script-based simulation tools are also more suitable for problems that use high-performance-computing or cloud computing on remote machines.

In the following sections we compare the simulation capabilities and use cases of SCAPS-1D and Solcore. These have been chosen for greater discussion as they provide contrasting approaches: SCAPS-1D is a well-established device level simulation package that is widely used in the experimental community, whilst Solcore is a highly modular, Python-based extensible open-source package that aims to provide flexibility for those who wish to develop and extend the code for their own research purposes.

### 6.3 Solar Cell Capacitance Simulator-1D

SCAPS (Burgelman et al., 2000) (a Solar Cell Capacitance Simulator) is one of the most widely used software tools for simulating solar cell device characteristics. (Verschraegen and Burgelman, 2007; Mostefaoui et al., 2015; Simya et al., 2015; AlZoubi and Moustafa, 2019; Karthick et al., 2020; Houimi et al., 2021; Jannat et al., 2021).

It was originally developed at the turn of the millenium for polycrystalline cell structures of the CIGS and CdTe family of materials, and is maintained by researchers based at the Department of Electronics and Information Systems (ELIS) of the University of Gent, Belgium. (Burgelman et al., 2000). It is designed to accommodate multiple thin films and interfaces, and has evolved over the years to include additional mechanisms for recombination (for example, Auger recombination) and tunnelling (for example, tunnelling at interface defects). As is common with the other software packages listed in Table 1, SCAPS-1D solves Poisson’s equation along with the continuity equations to describe carrier transport physics and calculate quantum efficiency (QE) and J-V characteristics. It can be used to describe imperfect materials through the specification of various parameters including recombination and capture rates at defect centres, and defect densities. This provides quantative predictions for the recombination current and points to which recombination mechanisms in a device a particularly limiting to device performance.

The primary advantage of SCAPS-1D is in terms of accessibility. SCAPS-1D is fully self-contained and free to download, is straight-forward to install on a modern operating system and is distributed with a graphical user interface so that those without any familiarity with programming can use it with ease. The lightweight (in terms of computational expense) and accessible nature of the programme, yet ability to model multiple thin-film layers and interfaces, has led to it being applied in several studies of potential interface materials. For example, Campbell *et al.*(Campbell et al., 2020) use SCAPS to compare CdS and In_2_S_3_ as the buffer layer in a CZTSSe cell. Combined with optical and electronic characterisation techniques they establish that although In_2_S_3_ has the more favourable band alignment, the presence of interfacial defect states results in a lower overall V_OC_.

### 6.4 Solcore

Solcore is a complete semiconductor solver written in Python 3 and developed by researchers at Imperial College London. (Führer et al., 2013; Alonso-Álvarez et al., 2018; Nikander et al., 2020).

The packages are arranged in a modular manner, with modules dedicated to materials science (including a material parameters database and quantum solver), optical methods, light sources, solar cell calculators and a large-circuit solver. Together these modules provide optical and electronic solving capabilities for a wide range of solar cell materials, with specific attention placed on III-IV devices, such as GaAs, and semiconductor nanostructures where quantum confinement effects are dominant. As such, Solcore is a multi-scale tools that extends from the micron length scales of light propagation to module-scale performance characteristics.

Device characteristics in Solcore can be predicted using a range of techniques (detailed balance and the diode equation), the most accurate being PDD as introduced above. A key advantage of Solcore is that it is designed for integration with external libraries. One such library is SMARTS (Gueymard, 1995) which incorporates a model of the atmospheric transfer of radiative sunshine. This allows for an accurate description of the solar irradiance depending on atmospheric conditions (such as water vapour, nitrogen dioxide, ozone or uniformly mixed gas absorption) along with other modelling capabilities such as Raleigh scattering, light diffusivity and back scattered light rays. Solcore is somewhat unique in that it has been designed with user extension and adaptation in mind: code development takes place in a public facing Github repository, the code has a modular structure, and Solcore ‘bootcamps’ have been organised for training and community building purposes. This is in contrast to the majority of simulation packages that are often high level, self-contained and do not invite extensive customisation.

Solcore has been applied to a number of research problems that incorporate multi-junction, quantum well and light-trapping technologies. For example, Pearce *et al.* use Solcore for the development of a perovskite on silicon tandem cell, where the perovskite top cell is deposited conformally onto the pyramidal surface of a silicon cell. (Pearce and Ekins-Daukes, 2019). Solcore has also been used in the development of ultra-thin GaAs cells for space applications. Here the challenge is to develop cells which are both physically thin (for radiation resistance) yet optically thick (for strong light absorption). Sayre *et al.* used the Solcore optical and solar cell modules to model and optimise these novel structures. (Sayre et al., 2022).

These examples demonstrate some important points about the role software design plays in enabling science. In the study from Pearce *et al.* the Rayflare software package used Solcore’s light source module and Structure class to calculate light absorption in a challenging geometry. This was made possible through the interoperability of each code, a feature which will become increasingly important in the quickly growing ecosystem of open-source tools for PV. (Holmgren et al., 2018c; Ayala Pelaez and Deline, 2020; Kim S et al., 2020; Silva et al., 2022). In addition, the development of PV devices for niche applications, such as high-efficiency and radiation-resistance solar cells for space, highlights the importance of code extensibility and adaptability. For example, researchers in the field of space PV may wish to extend a code to include physical models particularly relevant at ultra-thin length scales, such as carrier recombination at radiation induced defect sites.

Within both Solcore and SCAPS-1D there are assumptions that are made about the device so that the Poisson statistics and continuity equations can be used. For example, the solver in Solcore uses the Boltzmann approximation for carrier distribution with the assumptions that carrier concentrations are not highly doped, all carrier populations are in quasi-thermal equilibrium, and that the mobility of carriers are completely independent of the electric field. As a direct result of this latter assumption the Poisson drift diffusion model is only valid in weak electric fields. The various assumptions and model limitations inherent in all device modelling highlights the need for clear software documentation and on-going communication channels between software developers and users.

The accuracy of predictions made at the device level are always limited by the complexity of the atomic-scale interactions at the interface. For example, device models commonly assume that the work functions and electron affinity of the bulk materials govern the band bending observed on energy diagrams. However, as we have discussed earlier in this review, heterojunctions experience interface-specific bond formation, and this is especially true for polycrystalline materials that are prone to defect state formation at interfaces. (Raymond and Kronik, 2018). When the specifics of material-material interaction are not accounted for in device level models there will always be an inherent limitation to the accuracy of any predictions made.

## 7 Conclusion

As Herbert Kroemer famously wrote in his Nobel lecture, ‘The interface is the device’. (Kroemer, 2001). For solar cells, where charge transport across several heterojunction interfaces is a pre-requisite for working devices, this quote is particularly pertinent. However, despite material interfaces representing a bottleneck to the performance and stability of PV devices, their accurate simulation is less developed compared to bulk materials, and do not present the same accuracy, reliability or computational efficiency.

The main challenge for interface modelling is the inherent multi-scale nature of the problem. There is now the theoretical framework and computer power required to both build atomic scale structural interface models and predict the associated electronic properties. However, as explored towards the start of this review, the processes at an interface are often complex and intertwined, involving descriptions of chemical, thermal, electronic and thermodynamic behaviours. In this review we have discussed several approaches for modelling interface processes at various length and timescales, and have split these into three broad sections: data-driven approaches for high-throughput screening or lower-cost predictions of atomic-scale properties, atomistic approaches for high-accuracy predictions and explicit modelling of the structural and electronic changes at an interface, and continuum level models for predictions of device behaviour. We have outlined cases where these approaches have enabled a better understanding of material or device performance, alongside their key limitations.

In response to the challenges facing interface modelling, there are an abundance of opportunities for the future. In particular, we see opportunities for the data-driven approaches developed for bulk materials to be extended to consider the interactions at the interface between two material. We also see opportunities for the increased development and use of software programmes that embrace the multi-scale nature of semiconductor device simulation, and that combine quantum solvers alongside optical modelling, electrical modelling and large circuit solvers. Finally, we note that the majority of the most popular device-level simulation tools are self-contained applications which are not designed for user extension to new models. Given the fast-paced and increasingly diverse nature of photovoltaic research and development, we believe continued investment into open-source multi-scale software tools that are accessible, adaptable and extensible by members of the research community will accelerate both our understanding of materials interfaces and, as a consequence, the design of high-performing photovoltaic technologies.

## Data Availability

No datasets were generated in this study.
